# Basic determinants of child linear growth outcomes in sub-Saharan Africa: a cross-sectional survey analysis of positive deviants in poor households

**DOI:** 10.1038/s41598-022-18568-z

**Published:** 2022-08-20

**Authors:** Dickson A. Amugsi, Zacharie T. Dimbuene

**Affiliations:** 1 Maternal and Child Wellbeing Unit, African Population and Health Research Center, APHRC Campus, PO Box 10787-00100, Nairobi, Kenya; 2 Department of Population Sciences and Development, University of Kinshasa, Kinshasa, Democratic Republic of the Congo; 3 Microdata Access Division, Statistics Canada, 100 Tunney Pasture Driveway, Ottawa, K1A 0T6 Canada

**Keywords:** Diseases, Risk factors, Nutrition, Public health, Weight management

## Abstract

Childhood malnutrition is a significant public health problem confronting countries across the globe. Although there is evidence of a downward trend in undernutrition globally, sub-Saharan Africa did not experience significant improvement in the past decades. This study investigated the basic determinants of linear growth among children living in poor households. We analysed a nationally representative sample of children aged 0–59 months (N = 24,264). The study countries were Ghana, Kenya, the Democratic Republic of Congo (DRC), Nigeria, and Mozambique. The child’s height-for-age Z-scores (HAZ), categorised into HAZ > − 2 standard deviations (SD) (not stunted) and HAZ < − 2 SD (stunted) was the outcome variable of interest. We used logistic regression as our analytical strategy. In DRC, Ghana, Kenya and Nigeria, maternal years of schooling was associated with positive linear growth among children living in poor households. In Ghana and DRC, four antenatal visits had a positive effect on better linear growth, while in Nigeria, healthy maternal body mass index (kg/m^2^) had a positive effect on child's linear growth. The putative socio-demographic determinants investigated in our study can promote the linear growth of children living in poor households. Interventions aimed at fostering linear growth among children living in poverty should focus on enhancing these factors.

## Introduction

Childhood undernutrition is a significant public health problem confronting countries across the globe. Nonetheless, the available evidence suggests that global trends in child malnutrition have improved over the years. It is estimated that childhood stunting (short stature for age), a critical undernutrition metric, decreased from 39.7% in 1990 to 26.7% in 2010^1^. The trend is predicted to reduce to 22% in the next decade^1^. Despite global-level progress in reducing the problem during the past decades^2^, Africa has not seen much improvement. There was a decline in the stunting trend from 40.5% in 1980 to 35.2% in 2000^3^, the progress stagnated at 40% between 1990 and 2010^1^. The level of decline in sub-Saharan Africa (SSA) is not different from the African region. In SSA, the stunting trends decreased from 43% in 2000 to 34% in 2018^4^. The risk factors contributing to the high stunting prevalence in Africa are well documented^5–9^.

The consequences of stunting on the later life of the child are well known. Substantial evidence suggests a strong association between stunting and long-term effects such as poor cognitive development, school achievement, and economic productivity in adulthood and maternal reproductive outcomes^10–13^. Given the negative consequences of stunting on child health outcomes, the international community has paid considerable attention to the problem. For instance, the World Health Assembly (WHA) Resolution (2012) set a 40% reduction in the number of stunted children under-5 as one of the six global nutrition targets for 2025^11,14^. The Sustainable Development Goals (SDGs) also captured this undernutrition metric as a critical developmental target^15^. The above discussion suggests the need for substantial investment in nutrition interventions to address childhood stunting, as averting stunting could produce life-long benefits. It is, therefore, imperative to illuminate the broader factors that promote the linear growth of children living in adversity to provide evidence for the design of effective nutrition interventions. These factors are identified by the modified UNCEF conceptual framework^16^ as proximal and distal determinants, and are found to have a graded effect on positive child growth and health outcomes. The present study is set out to provide this evidence by focusing on factors that foster the growth outcomes of children living in poverty rather than risk factors of child growth deficiencies.

The evidence further suggests a disproportionate burden of stunting among children in low and middle-income countries, which is attributable to poverty, lack of food, and high incidence of infectious diseases, among others^3,11,17,18^. In several SSA countries, the prevalence of stunting among children remains stubbornly high^3,11,18^. The problem is notably more severe among children living in poor households^18–20^—they tend to have the highest prevalence of childhood stunting^18^. It is the case because poverty creates conditions that favour poor child growth outcomes and prevents affected populations from obtaining adequate access to prevention and care^18^. Despite the health challenges facing children living in poverty, some children live in the same conditions *(positive deviants)* or, even worse yet, have positive growth outcomes comparable to children residing in privileged households anywhere in the world^21–24^. Thus, positive deviance (PD) is based on the observation that in “*every community there are certain individuals or groups whose uncommon behaviours and strategies enable them to find better solutions to problems than their peers, while having access to the same resources and facing similar or worse challenges”*^25,26^. This paper intends to investigate the following questions: How are some children in poor households not stunted, although they are faced with similar adversity as those who are stunted are? What are the possible factors that help them to have better growth outcomes? Understanding this will help design programmes to promote the growth of children in impoverished households or environments.

The concept of *positive deviance* (as referenced above) is based on the premise that even in places where poverty is severe and widespread, some families can cope and harness scant resources sufficient to support optimal child health^25,26^. The positive deviance (PD) approach is founded on the idea that problems can be overcome using solutions within the community^27,28^. PD often studies the behaviours and characteristics of individuals who have better health outcomes than their peers who live in the same environment^27^. The PD approach was used previously to investigate several health-related issues in diverse settings^29–37^. In statistical analysis, the approach is often quantified as those who do not suffer from a negative outcome of interest relative to those who live in the similar environment with same resources but experience the negative effect of the outcome of interest^27^. The PD approach helps to focus on the 'positive' aspects of an outcome instead of the 'negative' which may make it possible to identify potential intervention points. In the context of this study, children who live in poor households but did not suffer from linear growth deficiency are considered *positive deviants.* The current study was intended to investigate the socio-demographic factors associated with better child growth outcomes in poor households. This resource-focused approach moves away from the dominant risk model approach, where the focus is usually on risk factors of child growth deficiencies. Using the PD approach is to help understand the drivers of better child growth and interventions to promote these drivers in poor households effectively.

## Methodology

### Data sources and sampling strategy

We analysed the Demographic and Health Surveys (DHS)^38^ data of poor households from Ghana, Kenya, Nigeria, Mozambique, and the Democratic Republic of Congo (DRC). The selection of the five countries was informed by our previous analysis using the same countries and data^39–41^. The DHS collects nationally representative data every five years in lower- and middle-income countries^42,43^. The DHS utilises a two-stage sample design. A detailed description of the design and sampling strategies can be found elsewhere^44–48^. The DHS data collectors interviewed all eligible study participants in their respective households of each country using standardised questionnaires and interview protocols. In this analysis, we used data of children aged 0–59 months and their mothers aged 15–49 years living in poor households. The DHS obtained children's data through face-to-face interviews with their mothers. The length/height of the children was measured using an adjustable measuring board calibrated in millimetres. Recumbent length (lying down on the board) was measured for young children, while standing height was measured for older children. The height data were converted into Z-scores based on the 2006 WHO growth standards, taking into account the age and sex of the child^49^. The present study involved an analysis of a total sample of twenty-four thousand, two hundred and sixty-four (N = 24,264) from poor households with children under five years. All methods in the DHS were performed in accordance with the relevant guidelines and regulations. For example, the collection and processing of the child anthropometry data were based on WHO guidelines.

### Ethics and consent

To ensure that the study was conducted based on high ethical standards^50^, data collectors were trained on how to respect the rights of individuals participating in the study. During the data collection, study participants were informed of their right to determine whether they wanted to participate in the study or not. They were also informed of their right to abstain or withdraw their participation at any time without reprisal. The risks and benefits of the study and steps taken to mitigate potential risks were adequately explained to study participants. The DHS sought and obtained ethical approvals of its protocols from government recognised Ethical Review Committees of the respective countries. Besides, the ethical clearance was granted by the Institutional Review Board of ICF International, USA, before the surveys were conducted. Informed consent was obtained from the mothers of the study children before they were included in the study. The DHS Program permitted the authors to use the data. The data were wholly anonymised, and therefore, the authors did not seek further ethical clearance before their use.

### Outcome and explanatory variables

#### Outcome variables

In this analysis, we used the child height-for-age Z-scores (HAZ) as the primary outcome variable. We reclassified the child HAZ into not stunted (better growth) and stunted (poor growth). We defined children with HAZ above − 2 SD (HAZ > − 2SD)^41,49,51^ as having a better linear growth/not stunted and described in this study as *positive deviants.* Similarly, children who had HAZ below − 2 SD (HAZ < − 2) from the median HAZ of the WHO reference population^49^ were considered stunted or having poor growth^41^. It is significant to underscore that DHS data contained all the three indicators of child nutritional status: height-for-age z-scores (HAZ), weight-for-age z-scores (WAZ), and weight-for-height z-scores (WHZ). However, we opted for HAZ because it is a cumulative indicator of a child's nutritional status, and therefore more informative and appropriate for use in the PD analysis. The WHZ, on the other hand, reflects more recent processes often associated with acute food shortages and/or illnesses leading to weight loss. At the same time, the WAZ lies between HAZ and WHZ—a child who has poor HAZ is also likely to be underweight, so is a child who has poor WHZ.

We stratified the analysis by household wealth index (WI). The WI has been used severally as an indicator for measuring inequalities associated with health outcomes as well as expenditure and income among households^43,44,46,52^. The detailed discussion on how the DHS created the WI is well documented^42,43,46^. In the datasets, the WI is categorised into five quintiles: poorest, poor, middle, richer, and richest^42,43,46^**.** In this paper, we recoded poor and poorest into poor/worse-off households. We combined the poorest and poor households' wealth quintiles because the literature suggests that children in these households have similar health outcomes^18–20^. We restricted all the analyses to the data from poor households.

The use of PD as an analytical lens informed the decision to limit the analysis to poor households. The PD approach usually focuses on people who thrive in adversity. Since poverty is a widely recognised risk factor for poor child growth and health outcomes, we treated children who have better growth/not stunted despite living in poor or disadvantaged households as children thriving in the face of adversity. We also seek to identify the potential factors that made it possible for these children to have better growth outcomes. Indeed, the use of the PD approach would not have been possible if we had combined both worse-off/poor and better-off/rich households in the same analysis. Therefore, although our earlier paper^41^ used data from the same source as the present paper, the data used in the analysis are not the same. For example, whereas the data for the previous article were from rich/better-off households, the data for the present analysis came from poor households.

Similarly, both the current and the previous study^41^ (referenced above) utilised logistic regression as an analytical strategy, yet the theoretical focus of the two is different. The significance of the current paper is the use of the novel PD approach as an analytical lens^26^. It moves away from the risk model and focuses on positive health. Secondly, it also deviates from the usual practice of classifying poverty as a risk factor for poor child growth outcomes. This may mask potential differences in child health outcomes within this sub-group. Our analysis was intended to unmask these differences, and the possible factors accounting for the disparities among poor households in relation to child outcomes.

#### Analytical framework

The modified UNICEF conceptual framework underpinned our analysis^16,53^. This framework outlines how the various factors/determinants influence child survival, growth, and development at different levels. These factors are analysed in terms of immediate, underlying, and basic determinants. The immediate determinants are adequate nutrients intake and health, while the underlying determinants are food security, care for children and women, healthcare, and a healthy environment^53^. The underlying determinants either influence child health directly or through the immediate determinants. The basic determinants (socio-demographic factors), in turn, influence the underlying determinants. In this context, the basic determinants are described as "exogenous" factors, which influence a child's nutrition status through their effects on the intervening proximate/underlying determinants. Thus, the underlying determinants are endogenously determined by the exogenous determinants^41,54^. For example, the effect of an exogenous variable such as maternal education on child growth outcomes is through its impact on good child-caring practices, including high utilisation of health care services.

### Data analysis

The present empirical analysis focused mainly on the basic determinants (i.e. socio-demographic factors). The scientific basis for this type of analysis is well documented^41,54–57^. Besides, we included antenatal care (ANC) and breastfeeding practices^54^ in our multivariable models. It was vital to have these variables because they can inform policies, programmes, and interventions rather than changes in household dynamics^54^—the critical role policy, institutional and contextual settings play in improving the prevalence of breastfeeding practices is well documented^54,58^. Before deciding which variables to include in the regression models, we tested for multicollinearity, and none was found among the variables of interest.

In this analysis, we built two regression models for each of the five countries. In the first model, we included the various putative socio-demographic factors [maternal body mass index (BMI), education, age, work status, parity, breastfeeding practices, marital status, ANC, the gender of head of household, size of household, total number children under five years and place of residence]. Parity in this study refers to the number of times a woman has given birth to a fetus with a gestational age of 24 weeks or more^44^. We adjusted for child dietary diversity (DD)—the details of how the DD is created can be found elsewhere^39^, age, and sex in the second and final model^41^. The conceptual framework and the literature guided the selection of the explanatory variables^53^. We estimated the adjusted odds ratio (aORs) of the effects of the socio-demographic determinants on child growth in poor households. Since DHS utilised complex sample design (CSD), we adjusted for design effects in all the analyses.

## Results

### Characteristics of study samples

Table 1 presents the results of the descriptive analysis. The results showed that 76% of children in Ghana were not stunted, while Kenya reported 68%. The prevalence in Mozambique, DRC, and Nigeria, ranged from 50 to 52%. Twenty-four per cent (24%) of children in Mozambique achieved the minimum dietary diversity (consumed at least four out of 7 food groups). Conversely, a small proportion (6%) of children in the DRC and Nigeria achieved the minimum dietary diversity. Also, 85% of women in Mozambique had normal weight (BMI). The prevalence in Kenya, Nigeria, Ghana and the DRC ranged from 68 to 76%. Further, 23% of women in Ghana had secondary school education, while Mozambique had just 1.20% (the lowest). In all countries, less than 1% of the women included in our analysis had higher education. In the DRC 8 out of every 10 women used antenatal care services during the pregnancy of their most recent child, while in Ghana, 6 out of every 10 women accessed antenatal care and 2 out of every 10 women in Nigeria.

**Table 1:** Characteristics of the study variables of the five countries.

Variables	DRC (n = 3979)		Ghana (n = 1453)		Kenya (n = 4967)		Mozambique (n = 3487)		Nigeria (10,378)	
	%/mean	SD	%/mean	SD	%/mean	SD	%/mean	SD	%/mean	SD
**Child-level covariates**										
Height-for-age > − 2SD (positive deviance)	50.0		76.0		68.0		52.0		51.0	
DD < 4 food groups	94.0		90.6		88.5		76.4		93.9	
DD ≥ 4 food groups	6.0		9.4		11.5		23.6		6.1	
Sex of child										
Female	50.4		48.8		50.4		51.3		50.6	
Male	49.6		51.2		49.6		48.7		49.4	
**Mother-level covariates**										
Body mass index (BMI)										
BMI < 18.50	15.8		7.4		17.0		7.99		12.4	
BMI = 18.50–24.99	76.3		73.3		68.0		84.6		74.4	
BMI = 25–29.99	6.7		15.7		12.2		6.91		10.5	
BMI ≥ 30	0.40		3.48		2.83		0.03		2.17	
Education										
No education	30.6		54.0		34.4		52.6		75.3	
Primary education	50.4		22.8		55.6		46.3		17.0	
Secondary education	19.0		23.2		9.43		1.17		7.6	
Higher education	0.05		0.1		0.1		na		0.1	
Working status										
Not working	20.0		16.5		45.9		61.5		35.3	
IS working	79.9		83.3		54.0		38.5		64.4	
Parity	4.44	2.46	4.21	2.3	4.36	2.46	4.37	2.43	4.83	2.75
Is breastfeeding	73.5		65.0		64.4		68.7		63.9	
Marital status										
Not in union	11.4		11.1		13.7		14.3		3.2	
Married	65.2		69.4		81.2		68.8		95.9	
Cohabiting	23.3		19.5		5.1		16.9		0.1	
Number of antenatal visits ≥ 4	77.2		59.2		31.1		28.3		18.8	
**Household-level covariates**										
Sex of household head										
Household head is female	24.1		18.3		34.4		28.0		6.5	
Household head is male	75.9		81.7		65.6		72		93.5	
Household size	6.4	2.55	6.67	3.14	6.22	2.35	5.97	2.5	7.63	3.42
Number of children under 5	2.24	0.98	1.98	0.99	2.02	0.87	2.03	0.91	2.5	1.22
**Community-level covariates**										
Urban residence	9.32		11.6		15.2		9.19		8.1	

*DD* dietary diversity, *DRC* Democratic Republic of Congo, *SD* standard deviation.

### Multivariable results of the association between socio-demographic factors and better child growth

Tables 2, 3, 4, 5 and 6 examine the association between socio-demographic characteristics and positive linear growth outcomes among children living in poor households. The results showed that one year change in mother's education was associated with 1.03 (95% CI = 1.01, 1.07), 1.06 (95% CI = 1.01, 1.11), 1.03 (95% CI = 1.01, 1.05), and 1.08 (95% = 1.06, 1.10) increased odds of positive child growth in DRC, Ghana, Kenya and Nigeria, respectively. In the analysis of the DRC and Ghana data, children of mother who attended at least four antenatal visits were respectively, 1.32 (95% CI = 1.05, 1.67) and 1.67 (95% CI = 1.19, 2.33) times more likely to have better linear growth outcomes compared to children of mothers who attended less than four antenatal care services. No significant association was observed in the remaining three countries. In Kenya, compared to children of non-working mothers, children of working mothers had 23% reduced odds of better growth (aOR = 0.77, 95% CI = 0.66, 0.91). In Nigeria, Mozambique, and DRC, a positive association was observed between breastfeeding and better child growth. However, this significant association disappeared after the child level factors were introduced in the models. Children living in urban areas had 28% reduced odds of better growth (aOR = 0.72, 95% CI = 0.55, 0.95) in Mozambique, while increased odds were observed in Nigeria (aOR = 1.58, 95% CI = 1.33, 1.87). In Nigeria, healthy maternal weight (normal BMI) was associated significantly with increased odds (aOR = 1.24, 95% CI = 1.08, 1.43) of better linear growth. In Nigeria, a mother being overweight was associated with increased odds (aOR = 1.51, 95% CI = 1.24, 1.83) of better linear growth. In Mozambique, a unit change in household size was associated with increased odds (aOR = 1.05, 95% CI = 1.01, 1.10) of better child growth. In Nigeria, maternal parity was associated with 5% reduced odds of better child growth (aOR = 0.95, 95% CI = 0.92, 0.98). In all the countries except Ghana, child level biological factors such as sex and age were associated with reduced odds of better linear growth.

**Table 2:** Multivariable analysis of the effects of socio-demographic factors on better linear growth/non-stunting among children living in poor households in DRC.

Variables	Model 1	Model 2
**Mother-level covariates**		
BMI (kg/m^2^) = 18.50–24.99	0.972 (0.758–1.247)	0.979 (0.753–1.274)
BMI (kg/m^2^) = 25–29.99	0.913 (0.604–1.379)	0.861 (0.551–1.348)
BMI (kg/m^2^) ≥ 30	0.310* (0.0795–1.207)	0.301 (0.0558–1.620)
Maternal education (in single years)	1.030** (1.001–1.060)	1.034** (1.003–1.065)
Age of the mother (in years)	0.994 (0.972–1.016)	1.012 (0.988–1.037)
Working status = is working	0.840 (0.671–1.052)	0.873 (0.684–1.113)
Parity	0.995 (0.934–1.060)	0.989 (0.923–1.061)
Is breastfeeding = YES	1.379*** (1.110–1.712)	0.813* (0.637–1.036)
Marital status = married	0.880 (0.648–1.196)	0.937 (0.677–1.297)
Marital status = cohabiting	0.986 (0.704–1.379)	1.036 (0.731–1.469)
Number of antenatal visits = 4 + visits	2.125*** (1.710–2.641)	1.321** (1.046–1.668)
**Household-level covariates**		
Head of HH is male	0.957 (0.759–1.206)	0.936 (0.741–1.183)
Household size	1.014 (0.965–1.065)	1.005 (0.954–1.058)
Number of children under 5 years	1.018 (0.904–1.147)	1.068 (0.943–1.210)
**Community-level covariates**		
Urban residence = urban	0.989 (0.740–1.322)	0.972 (0.720–1.312)
**Child-level covariates**		
Dietary diversity (DD) ≥ 4		1.049 (0.686–1.602)
Age of the child (in months)		0.959*** (0.953–0.965)
Sex of child = male		0.773*** (0.639–0.935)
Observations	**3979**	**3979**

Significant values are in bold.

95% confidence intervals (CIs) in parentheses.

*DD* dietary diversity, *HH* household, *BMI* Body mass index.

***p < 0.01, **p < 0.05, *p < 0.1.

**Table 3:** Multivariable analysis of the effects of socio-demographic factors on better linear growth/non-stunting among children living in poor households in Ghana.

Variables	Model 1	Model 2
**Mother-level covariates**		
BMI (kg/m^2^) = 18.50–24.99	0.944 (0.554–1.608)	0.946 (0.554–1.617)
BMI (kg/m^2^) = 25–29.99	1.702 (0.888–3.262)	1.727* (0.902–3.305)
BMI (kg/m^2^) ≥ 30	2.048 (0.739–5.677)	2.183 (0.783–6.089)
Maternal education (in single years)	1.059** (1.012–1.107)	1.057** (1.010–1.106)
Age of the mother (in years)	1.004 (0.966–1.044)	1.009 (0.969–1.050)
Working status = is working	0.772 (0.509–1.170)	0.798 (0.524–1.214)
Parity	1.009 (0.902–1.129)	1.007 (0.899–1.129)
Is breastfeeding = YES	1.705*** (1.206–2.410)	1.412* (0.975–2.045)
Marital status = married	1.316 (0.761–2.278)	1.300 (0.755–2.239)
Marital status = cohabiting	0.994 (0.539–1.831)	0.950 (0.516–1.747)
Number of antenatal visits = 4 + visits	2.004*** (1.464–2.743)	1.667*** (1.193–2.329)
**Household-level covariates**		
Head of HH is male	0.889 (0.549–1.440)	0.911 (0.566–1.468)
Household size	0.987 (0.928–1.050)	0.983 (0.923–1.047)
Number of children under 5	0.946 (0.774–1.156)	0.942 (0.771–1.150)
**Community-level covariate**		
Urban residence = urban	1.239 (0.735–2.087)	1.224 (0.733–2.046)
**Child-level covariates**		
Dietary diversity (DD) ≥ 4		1.281 (0.765–2.146)
Age of the child (in months)		0.989* (0.979–1.000)
Sex of child = male		0.850 (0.624–1.159)
Observations	**1453**	**1453**

Significant values are in bold.

95% confidence intervals (CIs) in parentheses.

*DD* dietary diversity, *HH* household, *BMI* body mass index.

***p < 0.01, **p < 0.05, *p < 0.1.

**Table 4:** Multivariable analysis of the effects of socio-demographic factors on better linear growth/non-stunting among children living in poor households in Kenya.

Variables	Model 1	Model 2
**Mother-level covariates**		
BMI (kg/m^2^) = 18.50–24.99	0.894 (0.716–1.116)	0.896 (0.716–1.122)
BMI (kg/m^2^) = 25–29.99	1.234 (0.910–1.674)	1.255 (0.923–1.706)
BMI (kg/m^2^) ≥ 30	0.982 (0.580–1.663)	0.971 (0.579–1.630)
Maternal education (in single years)	1.028** (1.006–1.051)	1.029** (1.006–1.052)
Age of the mother (in years)	1.026*** (1.006–1.046)	1.029*** (1.009–1.050)
Working status = is working	0.760*** (0.646–0.896)	0.774*** (0.656–0.914)
Parity	0.965 (0.911–1.023)	0.962 (0.907–1.020)
Is breastfeeding = YES	1.324*** (1.116–1.571)	1.182* (0.978–1.429)
Marital status = married	0.994 (0.777–1.270)	1.019 (0.796–1.303)
Marital status = cohabiting	0.951 (0.639–1.417)	0.967 (0.647–1.443)
Number of antenatal visits = 4 + visits	1.288*** (1.084–1.531)	1.173* (0.978–1.407)
**Household-level covariates**		
Head of HH is male	1.041 (0.873–1.242)	1.037 (0.868–1.239)
Household size	0.965* (0.924–1.007)	0.959* (0.919–1.002)
Number of children under 5	0.976 (0.878–1.084)	0.984 (0.884–1.095)
**Community-level covariate**		
Urban residence = urban	1.111 (0.900–1.373)	1.111 (0.898–1.375)
**Child-level covariates**		
Dietary diversity (DD) ≥ 4		0.914 (0.720–1.161)
Age of the child (in months)		0.991*** (0.987–0.996)
Sex of child = male		0.717*** (0.615–0.836)
Observations	**4967**	**4967**

Significant values are in bold.

95% confidence intervals (CIs) in parentheses.

*DD* dietary diversity, *HH* household, *BMI* body mass index.

***p < 0.01, **p < 0.05, *p < 0.1.

**Table 5:** Multivariable analysis of the effects of socio-demographic factors on better linear growth/non-stunting among children living in poor households in Mozambique.

Variables	Model 1	Model 2
**Mother-level covariates**		
BMI (kg/m^2^) = 18.50–24.99	1.305* (0.968–1.760)	1.328* (0.985–1.789)
BMI (kg/m^2^) = 25.00–29.99	1.201 (0.792–1.821)	1.225 (0.806–1.863)
BMI (kg/m^2^) ≥ 30	1.503 (0.389–5.810)	1.528 (0.417–5.603)
Maternal education (in single years)	1.030 (0.990–1.072)	1.031 (0.990–1.073)
Age of the mother (in years)	1.012 (0.994–1.029)	1.017* (0.999–1.036)
Working status = is working	0.938 (0.798–1.102)	0.936 (0.795–1.102)
Parity	0.988 (0.935–1.045)	0.993 (0.939–1.050)
Is breastfeeding = YES	1.182* (0.991–1.411)	0.968 (0.798–1.173)
Marital status = married	0.941 (0.715–1.237)	0.923 (0.701–1.216)
Marital status = cohabiting	0.972 (0.710–1.330)	0.967 (0.706–1.326)
Number of antenatal visits = 4 + visits	1.182* (0.990–1.411)	1.001 (0.831–1.207)
**Household-level covariates**		
Head of HH is male	1.126 (0.912–1.390)	1.124 (0.910–1.388)
Household size	1.067*** (1.018–1.118)	1.053** (1.005–1.104)
Number of children under 5	1.006 (0.894–1.132)	1.040 (0.923–1.171)
**Community-level covariate**		
Urban residence = urban	0.709** (0.540–0.931)	0.721** (0.550–0.947)
**Child-level covariates**		
Dietary diversity (DD) ≥ 4		1.169 (0.968–1.413)
Age of the child (in months)		0.985*** (0.980–0.990)
Sex of child = male		0.743*** (0.635–0.870)
Observations	**3487**	**3487**

Significant values are in bold.

95% confidence intervals (CIs) in parentheses.

*DD* dietary diversity, *HH* household, *BMI* body mass index.

***p < 0.01, **p < 0.05, *p < 0.1.

**Table 6:** Multivariable analysis of the effects of socio-demographic factors on better linear growth/non-stunting among children living in poor households in Nigeria.

Variables	Model 1	Model 2
**Mother-level covariates**		
BMI (kg/m^2^) = 18.50–24.99	1.231*** (1.073–1.413)	1.241*** (1.080–1.428)
BMI (kg/m^2^) = 25.00–29.99	1.484*** (1.228–1.794)	1.508*** (1.243–1.828)
BMI (kg/m^2^) ≥ 30	1.216 (0.874–1.693)	1.221 (0.868–1.720)
Maternal education (in single years)	1.072*** (1.052–1.092)	1.076*** (1.056–1.096)
Age of the mother (in years)	1.012** (1.001–1.023)	1.018*** (1.007–1.030)
Working status = is working	1.056 (0.960–1.162)	1.081 (0.981–1.191)
Parity	0.952*** (0.925–0.979)	0.950*** (0.923–0.978)
Is breastfeeding = YES	1.320*** (1.199–1.453)	1.035 (0.933–1.149)
Marital status = married	0.900 (0.685–1.183)	0.923 (0.702–1.213)
Marital status = cohabiting	1.133 (0.646–1.987)	1.107 (0.622–1.970)
Number of antenatal visits = 4 + visits	1.354*** (1.201–1.525)	1.081 (0.955–1.224)
**Household-level covariates**		
Head of HH is male	0.955 (0.782–1.168)	0.953 (0.778–1.168)
Household size	0.991 (0.971–1.011)	0.986 (0.966–1.007)
Number of children under 5	1.017 (0.964–1.072)	1.040 (0.986–1.097)
**Community-level covariate**		
Urban residence = urban	1.518*** (1.283–1.796)	1.575*** (1.325–1.872)
**Child-level covariates**		
Dietary diversity (DD) ≥ 4		1.148 (0.941–1.401)
Age of the child (in months)		0.981*** (0.978–0.984)
Sex of child = male		0.843*** (0.771–0.923)
Observations	**10,378**	**10,378**

Significant values are in bold.

95% confidence intervals (CIs) in parentheses.

*DD* dietary diversity, *HH* household, *BMI* body mass index.

***p < 0.01, **p < 0.05, *p < 0.1.

## Discussion

The study examined the basic determinants (socio-demographic factors) associated with better child growth in poor households in five sub-Saharan African countries. We utilised a *positive deviance* approach as our analytical lens, whereby children who were not stunted though living in poor households were considered *positive deviants*. The findings showed that the effects of socio-demographic factors on child growth vary across countries. Maternal higher years of education had a significant positive effect on better linear growth among children living in poverty in DRC, Ghana, Kenya, and Nigeria. This finding suggests that maternal education could mitigate the adverse effects of poverty on children's nutritional status. Thus, education is an essential resource for improving child growth outcomes in the face of adversity. It is possibly the case because the UNICEF conceptual framework^16,53^ suggests a direct effect of maternal education on caring practices, adequate dietary intake, utilisation of health care service, and a healthy environment. The aforementioned proximate factors, in turn, have direct effects on positive child growth outcomes. The literature on the benefits of mothers' education on child-caring practices and the utilisation of health services and the consequential positive effect on child health outcomes abound^59–62^. Our study findings are consistent with the existing literature^41,63^. A study using data from three SSA countries showed that a higher level of maternal education was associated with reduced odds of child stunting^63^. Also, our recent analysis using data from better-off households demonstrated the importance of maternal education on child growth outcomes^41^. Relating this to the findings in the present paper suggests that the positive effect of maternal education on child growth outcomes is irrespective of whether a child lives in adversity or not. Consequently, the literature and the present study, though using slightly different analytical and theoretical lenses, demonstrated the importance of education in improving child growth outcomes.

Further, the results showed that in DRC and Ghana, mothers who attended at least four antenatal visits (ANC) have children with better linear growth outcomes. The findings in these two countries depart from our previous work^41^, where ANC did not influence child growth outcomes in any of the countries included in the analysis. The positive effects observed in the present investigation suggest that in adversity, mothers who can attend at least four ANC visits have children with better growth outcomes. This may imply that ANC attendance can offset the adverse effects of poverty on child growth outcomes. The positive effect of ANC could be because mothers who attend ANC are likely to receive health and nutrition education, which may positively impact caring practices, with a trickle-down effect on their children health outcomes^64^. The above explanation is in line with our conceptual framework, which posits that the proximate factors are pathways through which the exogenous factors influence child growth outcomes^53^. Our findings are similar to those of previous researchers. Kuhnt and Vollmer^65^ found that having at least four ANC visits was associated with a reduced risk of stunting in pre-school children. Therefore, any efforts to promote ANC attendance among women may have a beneficial effect not only on the mothers but also on their offspring. Therefore, interventions to promote child growth in poor environments should incorporate ANC as a critical intervention package.

The study also illuminated the widely recognised benefits of breastfeeding for improved child health and growth outcomes^66–68^, but only when socio-demographic factors were introduced in models. For example, in Ghana, Mozambique, and DRC, a statistically significant association was observed between breastfeeding and positive child growth outcomes in the model containing only the socio-demographic factors. However, after controlling for child level covariates (dietary diversity, age and sex), the effect of breastfeeding on child linear growth failed to reach statistical significance. This finding may mean that whether breastfeeding will have a positive effect on child growth or not depends to some extent on the inclusion or otherwise of child-level covariates. Therefore, when examining the effects of socio-demographic factors on child linear growth, it is critical to include child-level covariates to avoid presenting misleading estimates^69^. These findings are inconsistent with our previous analysis, where breastfeeding was found to have a significant positive effect on child growth outcomes^41^. It is important to point out that the non-significant association between breastfeeding and child growth has previously been documented^62,68,69^. Marquis and colleagues^68^ observed an inverse relationship between breastfeeding and child linear growth. They attributed this inverse relationship to what they termed *reverse causality*—that is, breastfeeding did not lead to poor growth, but poor growth and health led to increased breastfeeding.

Surprisingly, in Mozambique, the widely recognised urban advantage concerning favourable health outcomes was not observed in the present study. The analysis showed that urban place of residence was associated negatively with child linear growth in poor households. The inverse relationship could be attributed to the possible precarious conditions in which some urban poor reside^69^. In the literature, both negative and positive effects have been found with the urban residence and child growth outcomes^70,71^. The urban children are usually taller and heavier^70,71^. However, this may not include those children in poor urban settings, as there is evidence that children in these settings tend to have shorter heights than expected^70^.

### Strengths and limitations of the study

The study involved the analysis of nationally representative data, making it possible for the findings to be generalised at the national level. Further, using data from multiple countries helped to highlight differences and similarities in the effects of the various covariates on child linear growth outcomes between the countries included in the analysis. Another significant strength of this analysis is its focus on resources for positive linear growth of children living in poor households rather than treating poverty as risk factor for child growth deficiencies. A limitation worth mentioning is that the data used in the analysis are cross-sectional in nature and therefore no causality can be established between the predictor and outcome variables. Hence the conclusions in the paper are interpreted in the context of associations between the predictor and the outcome variables*.* The use of quantitative data to investigate the PD approach may be a bit limiting as it would not be possible to explore all PD behaviours quantitatively. This limitation Notwithstanding, PD is a well-established concept, making it possible to explore the approach (PD) using quantitative data.

## Conclusions

The study examined the effects of child, maternal, household, and community levels socio-demographic factors on better linear growth among children living in poor households in SSA. The results showed that the effects of socio-demographic factors on child linear growth vary across countries. Maternal education has a positive effect on better growth among children in all countries except Mozambique. Promoting girl child education in poor households may have a beneficial generational effect on child growth outcomes. A higher number of ANC visits has a significant positive effect on better child growth. Interventions to promote linear growth among children living in poverty should incorporate ANC as a critical intervention package.

## Data Availability

The datasets generated and/or analysed during the current study are available in the DHS program repository, https://dhsprogram.com/data/available-datasets.cfm. Data are accessible free of charge upon a registration with the Demographic and Health Survey program (The DHS Program). The registration is done on the DHS website indicated above.
